# Novel SNP improves differential survivability and mortality in non-small cell lung cancer patients

**DOI:** 10.1186/1471-2164-15-S9-S20

**Published:** 2014-12-08

**Authors:** Tzia Liang Mah, Xin Ning Adeline Yap, Vachiranee Limviphuvadh, Nanpu Li, Srinath Sridharan, Vellaisemy Kuralmani, Mengling Feng, Natalia Liem, Sharmila Adhikari, Wei Peng Yong, Ross A Soo, Sebastian Maurer-Stroh, Frank Eisenhaber, Joo Chuan Tong

**Affiliations:** 1 Data Analytics Department, Institute for Infocomm Research, 1 Fusionopolis Way, #21-01 Connexis South Tower, Singapore 138632, Singapore; 2 Department of Haematology-Oncology, National University Health System, 5 Lower Kent Ridge Road, Singapore 119074, Singapore; 3 Bioinformatics Institute, 30 Biopolis Street, #07-01 Matrix, Singapore 138671, Singapore; 4 School of Biological Sciences (SBS), Nanyang Technological University (NTU), 60 Nanyang Drive, 637551 Singapore; 5 School of Computer Engineering (SCE), Nanyang Technological University (NTU), 50 Nanyang Drive, 637553 Singapore; 6 Department of Biological Sciences (DBS), National University of Singapore (NUS), 8 Medical Drive 4, 117597 Singapore; 7 Department of Biochemistry, Yong Loo Lin School of Medicine, National University of Singapore, Singapore 117597, Singapore; 8 Institute of High Performance Computing, 1 Fusionopolis Way, #16-16 Connexis, Singapore 138632; 9 Cancer Science Institute of Singapore, National University of Singapore, Singapore

**Keywords:** Biomarker, Genetic variant, Mortality, Survival Outcome, Non-small cell lung cancer

## Abstract

**Background:**

Non-small cell lung cancer (NSCLC) is a major cause of cancer-related death worldwide due to poor patient prognosis and clinical outcome. Here, we studied the genetic variations underlying NSCLC pathogenesis based on their association to patient outcome after gemcitabine therapy.

**Results:**

Bioinformatics analysis was used to investigate possible effects of POLA2 G583R (*POLA2+1747 GG/GA*, dbSNP ID: rs487989) in terms of protein function. Using biostatistics, *POLA2+1747 GG/GA *(rs487989, POLA2 G583R) was identified as strongly associated with mortality rate and survival time among NSCLC patients. It was also shown that *POLA2+1747 GG/GA *is functionally significant for protein localization via green fluorescent protein (GFP)-tagging and confocal laser scanning microscopy analysis. The single nucleotide polymorphism (SNP) causes DNA polymerase alpha subunit B to localize in the cytoplasm instead of the nucleus. This inhibits DNA replication in cancer cells and confers a protective effect in individuals with this SNP.

**Conclusions:**

The results suggest that *POLA2+1747 GG/GA *may be used as a prognostic biomarker of patient outcome in NSCLC pathogenesis.

## Background

Non-small cell lung cancer (NSCLC) is a leading cause of cancer mortality worldwide with over one million deaths annually [1]. It accounts for 75% of lung cancer cases and consists of three major subtypes: adenocarcinoma, large-cell carcinoma, and squamous-cell carcinoma [2]. Recent introduction of targeted therapy and increasing numbers of available chemotherapeutic regimens, such as platinums, taxanes and gemcitabine, do not effectively cure NSCLC patients, with varied response towards treatment and occurrence of drug toxicity [3,4]. In addition, prognosis remains dismal in NSCLC patients albeit careful evaluation of clinico-pathological factors that determine patient response to therapy, such as tumor, nodes and metastasis (TNM) staging, performance status, gender and weight loss. The long-term survival rate is low with only 14% of patients surviving five years after diagnosis [5] and the risk for relapse is high.

Gemcitabine is a third generation chemotherapeutic agent that has shown activity in NSCLC. Preclinical studies have shown that the compound is a potent radiosensitizer, with response in stage III NSCLC [6]. Gemcitabine can be administered as a single agent, or in platinum and non-platinum combination. The agent can also be combined with the chemotherapy drug pemetrexed, as well as the vascular endothelial growth factor (VEGF) inhibitor, for adenocarcinoma NSCLC. Due to its significant benefit and advantageous toxicity profile, gemcitabine has since evolved to become one of the most commonly used agents for lung cancer chemotherapy.

In recent years, much effort has been expended to identify genetic determinants in patient outcomes, so as to improve clinical treatment decisions and for the design of therapeutic agents. The epidermal growth factor receptor (EGFR) mutations, for instance, are common in patients with NSCLC [7], and are known to confer survival benefit and better clinical outcome when treated with EGFR tyrosine kinase inhibitors (TKIs) [8,9]. To date, no known genetic variants have been reported, that could help determine the dose and clinical outcomes in NSCLC patients receiving gemcitabine chemotherapy.

Here, we studied the polymorphism of genes involved in gemcitabine transport, metabolism and activity, based on their association to patient outcome after gemcitabine therapy. We showed for the first time that the single nucleotide polymorphism (SNP) *POLA2+1747 GG/GA *(rs487989) is a key determinant of mortality and survival outcome in gemcitabine-treated NSCLC patients. The *POLA2 *gene encodes DNA polymerase alpha subunit B in humans, which is involved in the initiation of chromosomal DNA replication [10-12]. The SNP causes DNA polymerase alpha subunit B to localize in the cytoplasm instead of the nucleus. This inhibits DNA replication in cancer cells and confers a protective effect in individuals with this SNP. The results suggest that *POLA2+1747 GG/GA *(rs487989) may be used as a prognostic biomarker of patient outcome in NSCLC pathogenesis.

## Results and discussion

### Association of genotypes and the mortality of NSCLC patients after gemcitabine therapy

How genetic variations affect the survival outcome of NSCLC patients after gemcitabine therapy is im-portant for improved clinical treatment decisions and for the design of therapeutic agents. Using Fisher's exact probability test and chi-squared test, we show, for the first time, that *POLA2+1747 GG/GA *(rs487989) is the most statistically significant SNP to be associated with mortality (Table 1), with a P value of 0.0406. The *POLA2 *gene corresponds to the p68 subunit of mouse DNA polymerase alpha, which couples the catalytic subunit of polymerase alpha to the primases, and translocates the polymerase alpha/primase complex from the cytoplasm to the nucleus for chromosomal DNA replication [13].

**Table 1:** Association between the 21 SNP genotypes and mortality of 43 NSCLC patients receiving gemcitabine, based on the corresponding P value.

No	SNP genotypes	Significance Level (P value)
1	*POLA2+1747 GG/GA*	0.0406

2	*RRM1(-756) TT/TC*	0.0896

3	*RRM1(-269) CC/CA*	0.0993

4	*SLC28A2+65 CC/CT*	0.1794

5	*SLC28A2+225 CC/CA*	0.1927

Fisher's exact probability test and chi-squared test are used. Only the top 5 ranked SNP genotypes are shown.

### *POLA2+1747=GG/GA *imposes differential effects on mortality and survival time of NSCLC patients after gemcitabine therapy

Using conditional probability test, we studied the effects of this *POLA2 *variant on mortality of patients after gemcitabine therapy. We found that the probability of death (P value = 0.0128) for patients with wild-type GG genotype is significantly higher (89.19%) than those with GA variant (50%).

**Figure 1:**
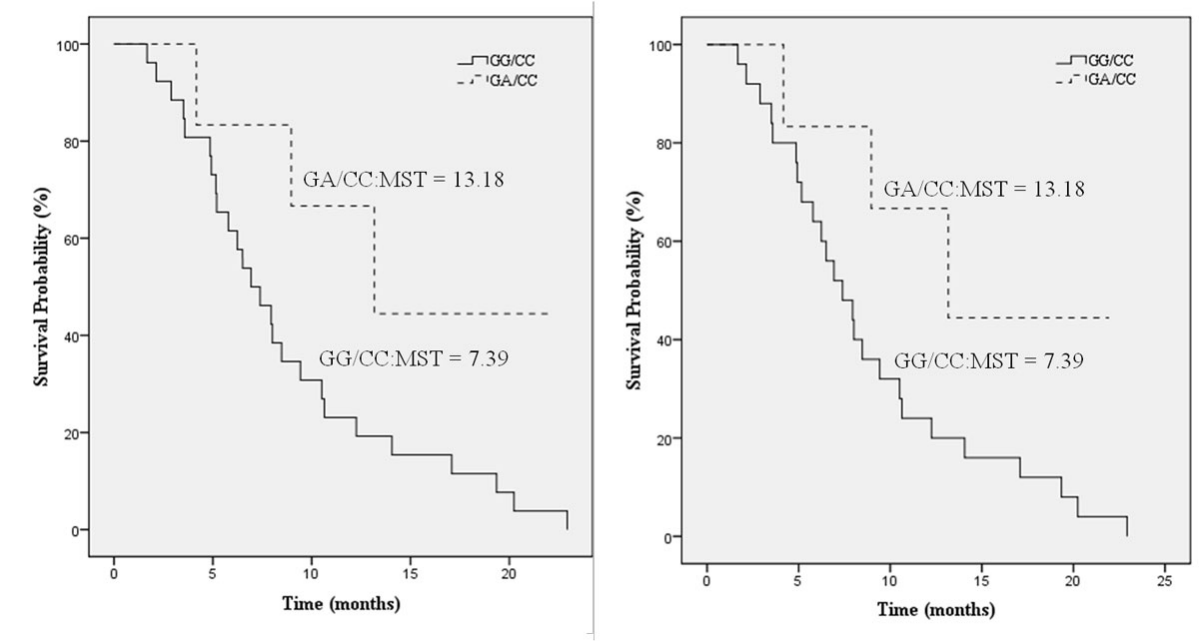
**Kaplan Meier plots**. (a) *POLA2+1747 GG/GA *and *SLC28A2+65 CC*, and (b) *POLA2+1747 GG/GA *and *SLC28A2+225 CC *showing the association of *POLA2+1747 GG/GA *with overall survival time.

Next, we studied the interactions among these 21 non-synonymous SNPs and how they impact the overall survival time of NSCLC patients. Table 2 shows the top ranked SNP-SNP interactions (based on their P value) that are associated with overall survival times of NSCLC patients. Among them, statistically significant differences in survival time between wild-type GG and variant GA genotypes are observed in *POLA2+1747 GG/GA *together with *SLC28A2+65 CC *(P value = 0.0004), and *POLA2+1747 GG/GA *together with *SLC28A2+225 CC *(P value = 0.0010). It is noteworthy that all the top-ranked SNP-SNP interactions listed in Table 2 involved POLA2. This clearly emphasizes the im-portance of POLA2 as a potential biomarker. These significant differences in survival time are depicted in Kaplan-Meier plot as shown in Figure 1. Patients with *POLA2+1747=GA *variant exhibit improved overall survival, as compared to their GG counterparts.

**Table 2:** Association between *POLA2+1747 GG/GA *interaction pairs and the overall survival time of 43 NSCLC patients receiving gemcitabine, based on the corresponding p-value.

No	Genotype Interaction Pairs		Significance Level (P value)
1	*POLA2+1747 GG/GA*	*SLC28A2+65 CC*	0.0004

2	*POLA2+1747 GG/GA*	*SLC28A2+225 CC*	0.0010

3	*POLA2+1747 GG/GA*	*RRM1(-756) TT*	0.0627

4	*POLA2+1747 GG/GA*	*SLC28A3+338 AA*	0.2859

5	*POLA2+1747 GG/GA*	*RRM1(-269) CC*	0.3767

Log-rank test and chi-squared test are used. Only the top 5 ranked genotype interaction pairs are shown.

*POLA2+1747 GA *together with *SLC28A2+65 CC *are observed to be associated with increased median survival time (Figure 1A). For *POLA2+1747 GG/GA *together with *SLC28A2+65 CC*, the median overall survival time of patients is 7.39 months and 13.18 months in patients with GG and GA genotypes, respectively (P value = 0.0004). Likewise, we also observed that the *POLA2+1747 GA *together with *SLC28A2+225 CC *are associated with increased median survival time (Figure 1B). For *POLA2+1747 GG/GA *together with *SLC28A2+225=CC*, the median overall survival time of patients with the wild-type GG genotype was 7.39 months and for GA variant is 13.17 months (P value = 0.0010) (Figure 1B).This result indicates that the non-synonymous *POLA2+1747 GA *SNP is an important interactor associated with increased survival time.

### Computational prediction of functional effects of POLA2 G583R

**Figure 2:**
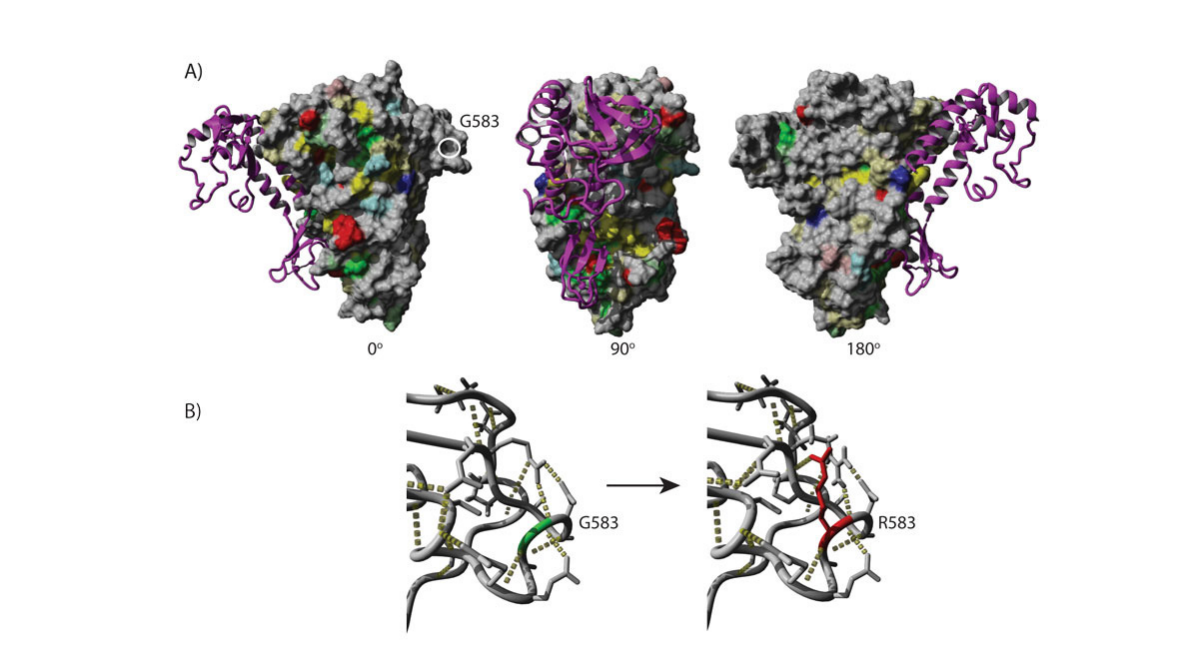
**Structural model and conservation mapping of human POLA2**. A) shows the POLA2 model in complex with the crystal structure of the carboxyl-terminal domain of DNA polymerase alpha (in purple) represented in different rotations. Conservation was mapped to the surface of the POLA2 model. Grey color means no conservation, while the other colors signify conservation of physical properties, i.e. yellow: hydrophobic, green: uncharged polar, blue: positive charge, red: negative charge. Color intensity is proportional to strength of conservation. B) shows side chain of amino acid at the SNP position including nearby area before and after change from glycine to arginine. G583 shown in green and R583 shown in red.

PolyPhen-2 [14] predicted POLA2 G583R (rs487989) to be "possibly damaging" with a score of 0.474 (sensitivity: 0.89; specificity: 0.90) and SNAP (Bromberg et al., 2008) suggested it to be "non-neutral". In contrast, when we used either the rsid or our alignment of orthologues (Additional File 1: Fig S1) as input for SIFT (Ng and Henikoff, 2001), the prediction was both "tolerated" with scores 0.45 and 0.50, respectively (score threshold <0.05 for "deleterious"). Figure 2A shows the homology model of POLA2 in complex with the carboxyl-terminal domain of DNA polymerase alpha (as seen in the yeast crystal structure). The SNP is located far from the evolutionary widely conserved interaction surface of the catalytic subunit in an opposite surface loop. This region is not particularly conserved among remote orthologues ranging from human to yeast and plants. Hence, the functional importance, if any, would be restricted to a subset of species more closely related to human. When we used FoldX (Schymkowitz et al, 2005) to predict the effect of the SNP on protein structure stability, interestingly, the average free energy change of the SNP was significantly elevated (3.79 kcal/mol, SD = 0.31) which means that the SNP has a destabilizing effect on the protein structure (Figure 2B). Since this result was derived from an energy minimized but static homology model, we wanted to see if this effect can also be reproduced in a dynamic model through molecular dynamics simulations. Indeed, 5 repetitions each of wildtype and mutant POLA2 simulations over 10ns in explicit water showed that the surface loop region harbouring the mutation is consistently destabilized and more flexible through G583R Additional File 2: Fig S2 and Additional File 3: S3). In the absence of presumably catalytic residues around the mutation site, the surface-exposed nature and taxon-restricted conservation of the loop would suggest a possible role for this region in protein interactions. Based on our structural modelling and simulations, the altered conformation and increased flexibility through the G583R mutation could potentially disrupt protein interactions. It is known that complex formation with various partners can influence POLA2 nuclear shuttling. Therefore, we hypothesized that a possible functional effect of G583R could be altering POLA2 localization in the cell.

### Subcellular localization of wild type POLA2 and mutant POLA2 G583R proteins

**Figure 3:**
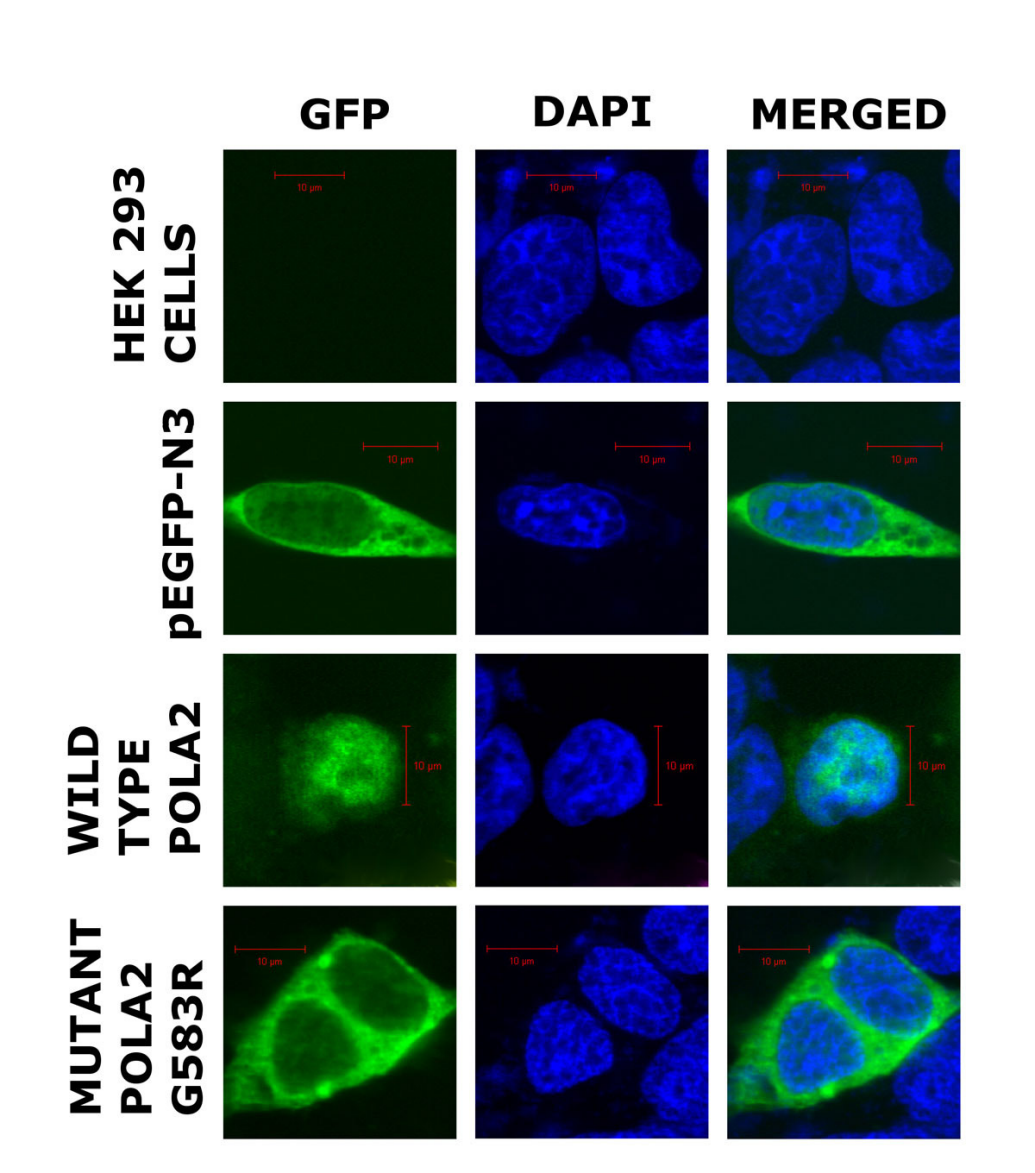
**Subcellular localization of GFP-tagged POLA2 wild type and mutant G583R proteins**. HEK 293 cells were transfected with GFP-fused proteins (green) as indicated and treated with anti-GFP followed by Alexa 488 (green) to stain the proteins and 4',6-diamidino-2-phenylindole (DAPI) (blue) to stain the nuclei and then examined by laser fluorescence confocal microscopy. The fields shown were visualized independently at the appropriate wavelength for anti-GFP (488 nm) and DAPI (405 nm), and then the two images were merged. Magnification: 63×. Scale bar is 10 µm.

Subcellular localization of proteins can help elucidate functional changes between wild type and mutant *POLA2+1747*. *POLA2+1747 GG/GA *SNP encodes for a glycine to arginine amino acid change (G583R) in the mutated POLA2 protein (POLA2 G583R). To study the subcellular distribution of the mutant and wild type DNA polymerase alpha subunit B, we constructed GFP fusion proteins and transfected HEK 293 cells. Untransfected HEK 293 cells were used as mock control to check the efficiency of the transfection reagent or other nonspecific effects. HEK 293 cells transfected with empty *pEGFP-N3 *vector were used to check the localization of GFP fusion proteins. To ensure that these proteins were successfully expressed in HEK 293 cells after transfection, we developed western blots containing HEK 293 cell lysate proteins and immunostained the blots with Mouse anti-GFP, followed by Goat anti mouse IgG-HRP. No protein band is observed for untransfected HEK 293 cells. Protein band size of about 27 kDa is observed for GFP control. On the other hand, the lanes for GFP constructs containing mutant POLA2 G583R and wild type POLA2 show us the expected protein band size of about 97 kDa each (27 kDa for GFP vector alone plus 70 kDa for the mutant and wild type DNA polymerase alpha subunit B). This indicates that the proteins are successfully expressed in the transfected cells. The subcellular localization of the mutant and wild type DNA polymerase alpha subunit B was studied with microscopy (Figure 3). Untransfected HEK 293 cells show no green fluorescence from GFP. For the pEGFP-N3 control, the green fluorescence protein is predominantly localized in the cytoplasm. We find that wild type DNA polymerase alpha subunit B is localized in the nucleus (DAPI-stained), whereas, the DNA polymerase alpha subunit B mutant (POLA2 G583R) is predominantly localized in the cytoplasm.

## Discussion

In a recent study on genes involved in gemcitabine pharmacology in ethnic Asian populations[15], we reported on the use of a statistical approach to examine associations between genotypes and the outcome of NSCLC patients including response rate, time to progression, gemcitabine toxicity and overall survival. We have now extended the study to another aspect of NSCLC patient outcome that was not examined previously, i.e. mortality, and also shown here that the *POLA2+174 GG/GA *(rs487989) is strongly associated with mortality rate and survival time among NSCLC patients treated with gemcitabine. We have previously shown that this particular SNP by itself did not have a significant effect on survival time [15]. Now, we found that its interaction with *SLC28A2+65 CC *and *SLC28A2+225 CC *led to an increase in the overall survival times of NSCLC patients.

This *POLA2+1747 *variant (rs487989) is not only present in the European and African populations [16], but is also prevalent in the Asian population among Chinese, Indians and Malays [15]. This SNP encodes for a glycine to arginine amino acid change (G583R), where *G *is an ancestral allele, resulting inside chain polarity and charge reversal. Here, we showed, through biostatistics, that individuals with the ancestral allele *G *for *POLA2 *tend to have lower survival rates in NSCLC pathogenesis, compared to individuals with *GA *polymorphism. To unravel possible molecular mechanisms of functional effects of this mutation, we utilized multiple computational approaches based on evolutionary conservation, structural modelling and molecular dynamics simulations. Given its location in a surface loop of the structure and causing flexible rearrangements of this surface area, we hypothesized that it could disrupt protein interactions which may be important for subcellular localization. Indeed, we experimentally showed that this point mutation is functionally significant, leading to a change in localization that is likely to affect regulatory activity and induces better survival in NSCLC patients treated with gemcitabine. The wild type POLA2 that is known to facilitate nuclear DNA replication is predominantly found in the nucleus, whereas the mutant POLA2 G583R protein [12] that is strongly associated with better survival in NSCLC patients is mainly localized in the cytoplasm. DNA polymerase alpha subunit B is required for cell viability [12]. By localizing in the cytoplasm, nuclear DNA polymerase alpha activity is inhibited. This confers a protective effect in NSCLC patients who possess the *POLA2+1747 GG/GA *SNP genotype, as the tumour DNA could not replicate. This inhibits tumour cell proliferation, and ultimately results in tumour cell death.

## Conclusions

In summary, we established that the *POLA2+1747 GG/GA *(rs487989) is a genetic determinant of clinical outcomes in NSCLC patients receiving gemcitabine treatment. EGFR mutations are used for profiling NSCLC patients treated with EGFR tyrosine kinase inhibitors, and similarly, the findings in this article can become a stepping stone for the discovery of new options for gemcitabine-based therapy. Due to the lack of genetic variants that could help determine the dose and clinical outcomes in NSCLC patients receiving gemcitabine chemotherapy, such biomarkers would be useful for doctors in treating patients more efficiently to achieve satisfactory clinical outcome and better survival.

## Materials and methods

### Study population

The study population consists of 43 NSCLC Chinese patients from our previous study [15]. Table 3 gives more details about the study population used in this work.

**Table 3:** Summary of the study population used in this work.

Total number of patients	43
Male patients	76.7%

Response rate	48.8%

Median survival	9.43 months

Median time to progression	5.5 months

Median age	64 years

Range of age	39-74 years

### Selection of gene variant loci

Here, we assessed 21 non-synonymous SNPs in 9 genes involved in gemcitabine transport, metabolism and activity [15]. The SNPs are found in the respective gene variant loci, namely, *CDA+79 *(rs2072671)*, CDA+208 *(rs60369023)*, CDA+435 *(rs1048977)*, DCK+3122 *(rs3775289)*, DCK+36791 *(rs1803484)*, DCTD+315 *(rs4742)*, POLA2+1747 *(rs487989)*, RRM1-756*(rs11030918)*, RRM1-269 *(rs12806698)*, S28A1+419 *(rs2277576)*, S28A1+565 *(rs2290272)*, S28A1+709 *(rs8187758)*, S28A1+1368 *(rs2242048)*, S28A1+1528 *(rs2242047)*, S28A1+1561 *(rs2242046)*, S28A2+65 *(rs61637002)*, S28A2+225 *(rs1060896)*, S28A3+338 *(rs10868138)*, TYMS-100 *(rs34743033)*, TYMS-58 *(rs2853542)*, TYMS+15705 *(rs34489327).

### Statistical Analyses

Fisher's exact probability test was used to assess the relationship between each of the 21 SNPs and the mortality of 43 NSCLC patients based on the p-values between genotypes. Conditional probability of death given a geno-type of a SNP was used to characterize the differential effects on mortality. Chi-squared test was employed to confirm the significance (P value) of the difference between genotypes. Differences were considered statistically significant when the P value was less than 0.05. All statistical tests were two-sided. Kaplan-Meier method and log-rank test were used to compare overall survival time for interaction pairs. SPSS software version 14.0 (SPSS Inc., Chicago, IL) was used.

### Bioinformatics analysis

In order to investigate possible effects of POLA2 G583R (*POLA2+1747 GG/GA*, dbSNP ID: rs487989) in terms of protein function, we analysed the mutation with PolyPhen-2 version 2.2.2 using the rsid (rs487989) of POLA2 G583R as input, SNAP using the amino acid sequence of human POLA2 (RefSeq ID: NP_002680) as input (Bromberg et al., 2008) and SIFT using a curated alignment of orthologous sequences (Ng and Henikoff, 2001). Orthologous sequences of human POLA2 (RefSeq ID: NP_002680) were retrieved with the orthologue search in ANNOTATOR (Ooi et al, 2009). A multiple alignment was created using MAFFT with the L-INS-I algorithm (Katoh and Toh, 2008). After deleting sequence that had large gaps in Jalview (Waterhouse et al, 2009), we selected diverse organisms and 24 remaining sequences were compared.

Homology modelling of human POLA2 was performed with Modeller (Eswar et al, 2008) using the crystal structure of the carboxyl-terminal domain of yeast DNA polymerase alpha in complex with its B subunit (PDB:3FLO) (Klinge et al, 2009) as template. Then, the alignment containing 24 diverse orthologous sequences (mentioned above) was used to calculate the conservation on individual positions using the evolutionary trace algorithm (Lichtarge et al, 2003) and the level of residue conservation was mapped to its corresponding position in the model and visualized with YASARA (Krieger et al, 2004). Moreover, we used FoldX (Schymkowitz et al, 2005) with prior energy minimization using the RepairPDB function and 5 repetitions of the mutation stability change calculations to predict the SNP effect on protein structure stability. Lastly, we performed 5 wildtype and 5 mutant MD simulations over 10 ns in explicit water using the AMBER03 force field in YASARA (Krieger et al, 2004) following standard protocols to understand the effect of the SNP on protein structure flexibility.

### Isolation of total RNA from HEK 293 cell culture

HEK 293 cells were lysed directly in a 10 cm culture dish using TRIZOL® Reagent (Invitrogen, Carls-bad, CA, USA). Total RNA was isolated and used for further experiments only if the RNA was found intact by running on 1% denaturing agarose gel.

### Reverse transcription of total RNA and *POLA2 *gene amplification by PCR

Using SuperSc*ript™ *III One-Step RT-PCR System with Platinum® Taq High Fidelity (Invitrogen), total RNA was reverse transcribed into complimentary DNA (cDNA), followed by amplification of the wild type *POLA2 *using forward primer with Kpn1 restriction site; 5'-AAGGTACCATGTCCGCATCCGCCCAGCA-3' and reverse primer with BamH1 restriction site; 5'-AAGGATCCGATCCTGACGACCTGCACAGCA-3'. Amplicon size was verified by running the PCR product and GeneRuler™ 100 base pairs DNA ladder on 0.8% agarose gel.

### Cloning of *POLA2 *amplicon and sequence verification

*The POLA2 *amplicon was cloned into pGEM®-T easy vector (Promega, USA), and subsequently transformed into *Escherichia coli *DH5α bacteria. The plasmid DNA was extracted and purified using QIAprep Spin Miniprep Kit (QIAGEN, Germany). Next, the concentration and purity of plasmid DNA was measured using NanoDrop (Thermo Fisher Scientific, USA). The resultant plasmid was digested with EcoRI and ran on 0.8% agarose gel to identify recombinant clones. Integrity of the wild type *POLA2 *constructs was verified by sequencing. Point mutation was introduced into *POLA2 *using the XL QuikChange Site-Directed Mutagenesis Kit, and *POLA2+1747 GG/GA *were verified by sequencing. Wild type *POLA2 *and *POLA2+1747 GG/GA *were then cloned into *pEGFP-N3 *(Clontech, USA) at Kpn1 and BamHI restriction sites.

### Cell culture and transfection

HEK 293 cells were grown in Dulbecco's modification of Eagle's medium (DMEM) supplemented with 10% fetal bovine serum (FBS) and 1% PSG (penicillin/streptomycin/glutamine) on 6-well plate and maintained at 37°C and 5% CO_2_. HEK 293 cells were transiently transfected using Lipofectamine 2000 (Invitrogen), following the manufacturer's protocol. After 5 hours of transfection with *POLA2-GFP *constructs in Opti-MEM® I Reduced Serum Medium (Cat. No. 31985-062), 30% FBS was added into each well, and incubated at 37°C and 5% CO_2 _overnight. After 17-18 hours of transfection with *POLA2-GFP *constructs using Lipofectamine 2000 (Invitrogen), cells were lysed in 1× cell lysis buffer from Matchmaker™Chemiluminescent Co-IP System (Clontech) with 25× complete EDTA free protease inhibitor (Roche, Germany) and 100× Phenylmethylsulfonyl fluoride (Sigma-Aldrich®, USA).

### Western blotting

Equivalent volumes (20 µl) of cell lysates were loaded onto 8% SDS-PAGE gels to resolve proteins. Then, proteins were transferred onto PVDF membrane and blocked using 5% non-fat dry milk for 1 hour to reduce non-specific binding and incubated overnight at 4°C with mouse anti-GFP (Roche) at 1:2500 dilution in 0.5% non-fat dry milk followed by Goat anti mouse IgG-HRP (Santa Cruz Biotechnology, US) at 1:10000 dilution in 0.5% non-fat dry milk for 1 hour at room temperature. Immunoblots were developed using Amersham™ ECL™ Prime Western Blotting Detection Reagent (GE healthcare, Sweden), following the manufacturer's protocol.

### Confocal laser scanning microscopic analysis

After 17-18 hours of transfection with the *POLA2-GFP *constructs using Lipofectamine 2000 (Invitrogen), HEK293 cells grown on glass cover slips were fixed with 2% paraformaldehyde in PBS at room temperature. Slides were blocked at room temperature for 1 hour with 5% BSA in 0.1% Triton/PBS and then immunostained with mouse anti-GFP (Roche) at 1:100 dilution followed by the Alexa Fluor 488 donkey anti-mouseIgG (Invitrogen, Molecular Probes) (1:2000 dilution) at room temperature for an hour. Images were captured with Zeiss LSM Meta confocal inverted mi-croscope with a magnification of 63×.

## Competing interests

The authors declare that they have no competing interests.

## Authors' contributions

TLM conceptualized the project. TLM and JCT designed the study. Clinical analyses were done by NL^2^, WPY and RAS. Statistical analyses were done by VK, MF and NL^1^. Bioinformatics and literature analyses were done by VL, FE and SMS. Cellular localization experiments were done by XNAY, SA and FE. The manuscript was written by TLM, JCT, VL, FE, SS and SMS.

## Supplementary Material

Additional File 1 **Fig. S1**. Multiple alignment of human POLA2. Click here for file

Additional File 2 **Fig. S2**. MD simulation of the human POLA2 wildtype and mutant model. Click here for file

Additional File 3 **Fig. S3**. Details of per residue fluctuations and comparison of representative conformations. Click here for file
