# Supplementary material for: Novel SNP improves differential survivability and mortality in non-small cell lung cancer patients
Source: BMC Genomics. 2014 Dec 8;15(Suppl 9):S20. doi: 10.1186/1471-2164-15-S9-S20 (PMC4290611; doi:10.1186/1471-2164-15-S9-S20)
Supplement: Additional File 1 — Fig. S1. Multiple alignment of human POLA2. [file 1471-2164-15-S9-S20-S1.docx]

**
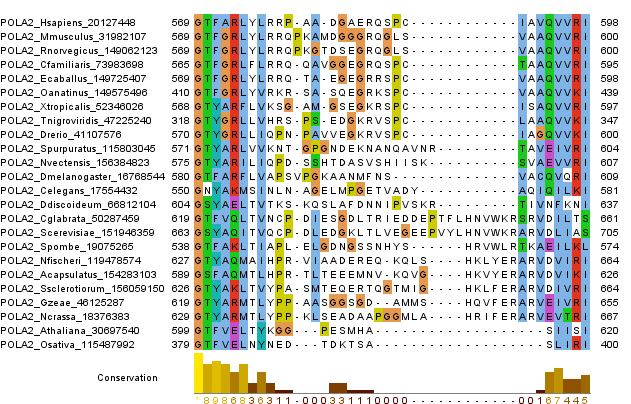
**

**Fig. S1. Multiple alignment of human POLA2.**

Orthologous sequences of human POLA2 (GI number: 20127448, RefSeq ID: NP_002680) were retrieved with the orthologue search in ANNOTATOR (Ooi et al., 2009). Multiple alignment was created using MAFFT with the L-INS-I algorithm (Katoh and Toh, 2008). After deleting sequence that had large gaps in Jalview (Waterhouse et al., 2009), we selected a phylogenetic diverse range of organisms and the 24 remaining sequences were compared. This Figure shows the alignment around the SNP position. The GI number of each orthologue can be found after the name of organism in the left side of the alignment.
