# Supplementary material for: Novel SNP improves differential survivability and mortality in non-small cell lung cancer patients
Source: BMC Genomics. 2014 Dec 8;15(Suppl 9):S20. doi: 10.1186/1471-2164-15-S9-S20 (PMC4290611; doi:10.1186/1471-2164-15-S9-S20)
Supplement: Additional File 2 — Fig. S2. MD simulation of the human POLA2 wildtype and mutant model. [file 1471-2164-15-S9-S20-S2.docx]

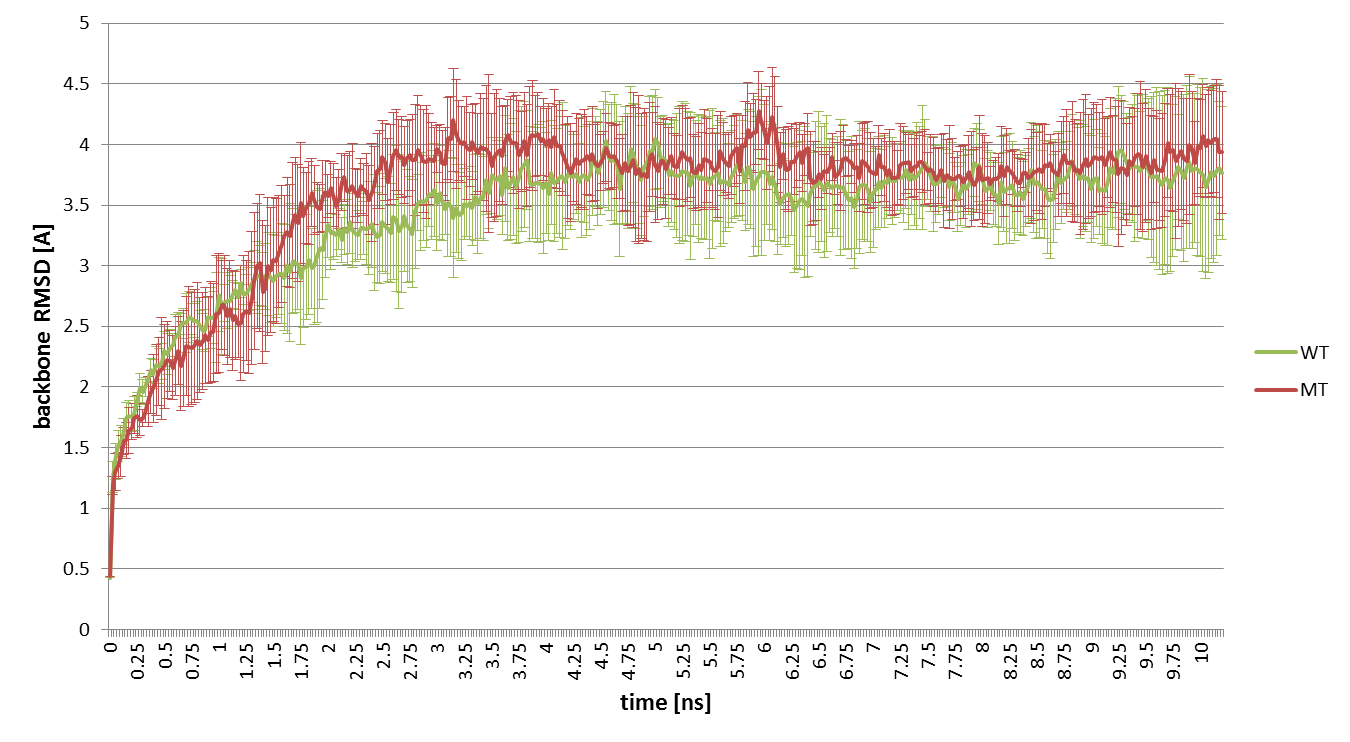


**Fig. S2. MD simulation of the human POLA2 wildtype and mutant model.**

5 wildtype and 5 mutant molecular dynamics (MD) simulations were run over 10ns in explicit water using the AMBER03 force field in YASARA to understand the effect of the SNP on protein structure flexibility. As seen by a higher backbone RMSD fluctuation after 2ns (when the simulation reached its equilibrium state), the G583R mutation causes destabilization through increased flexibility of the structure.
