# Supplementary material for: Novel SNP improves differential survivability and mortality in non-small cell lung cancer patients
Source: BMC Genomics. 2014 Dec 8;15(Suppl 9):S20. doi: 10.1186/1471-2164-15-S9-S20 (PMC4290611; doi:10.1186/1471-2164-15-S9-S20)
Supplement: Additional File 3 — Fig. S3. Details of per residue fluctuations and comparison of representative conformations. [file 1471-2164-15-S9-S20-S3.docx]

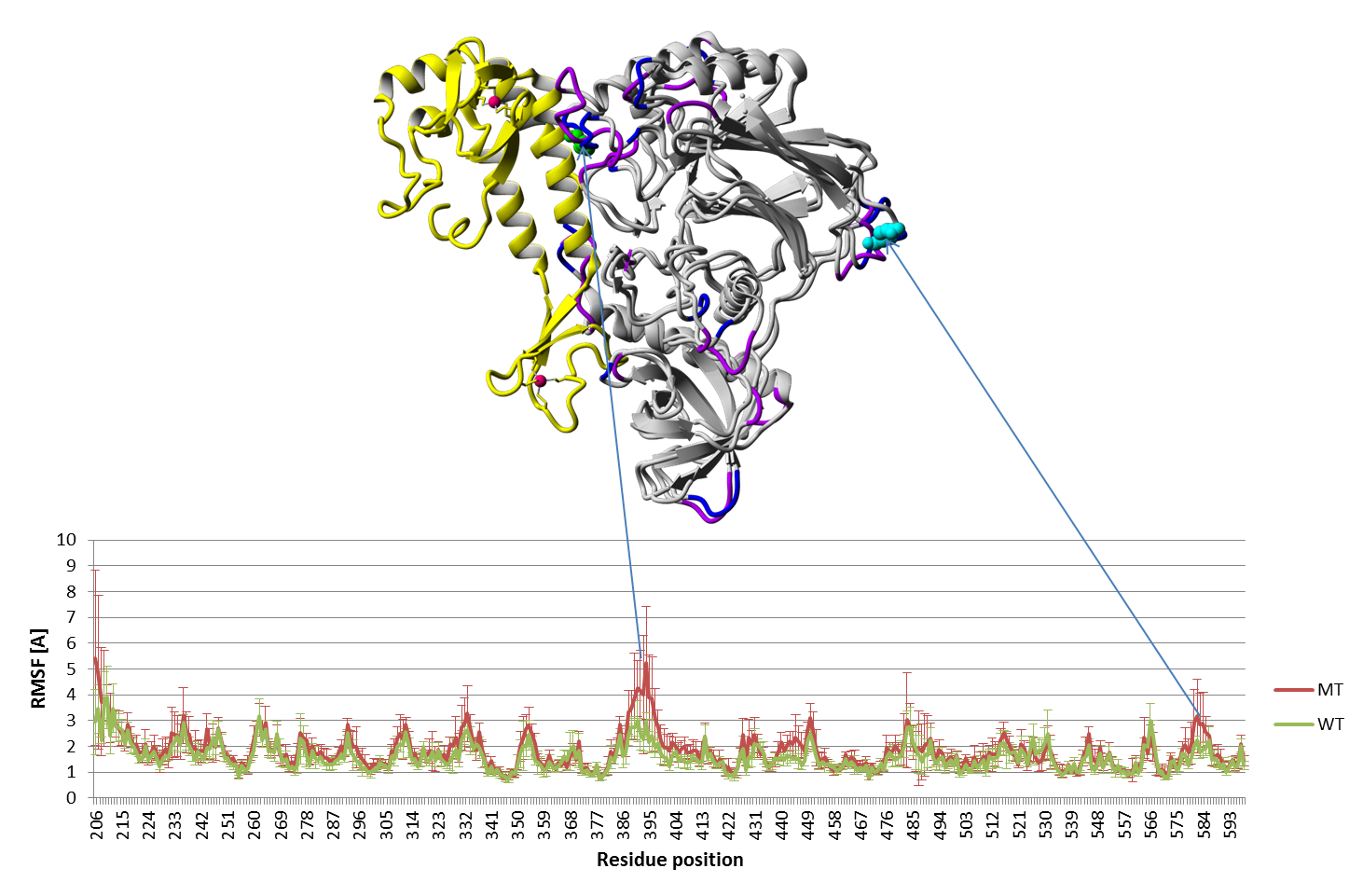


**Fig. S3. Detail of per residue fluctuations and comparison of representative conformations.**

Two major regions appear to fluctuate differently between the wildtype and mutant simulations (blue and purple in the grey POLA2 structure). As expected and predicted by the static FoldX calculations, the loop harboring the G583R mutations is also destabilized and more flexible in the dynamic simulations. Interestingly, we additionally observe an increased flexibility around L394 (POLA1 interaction site, POLA1 shown in yellow), although further studies are needed to confirm that this could be induced directly by the G583R mutation.
